# Comparison of three different multi-analyte point-of-care devices during clinical routine on a medical ICU

**DOI:** 10.1186/cc9552

**Published:** 2011-03-11

**Authors:** V Stadlbauer, S Wallner, T Stojakovic, KH Smolle

**Affiliations:** 1 University Hospital Graz, Austria; 2 Clinical Institute of Medical and Chemical Laboratory Diagnostics, Medical University of Graz, Austria

## Introduction

Multi-analyte point-of-care (POC) devices are important to guide clinical decisions in critical care. However, the use of different devices in one hospital might cause problems. We therefore evaluated three commonly used POC devices and analysed accuracy, reliability and bias.

## Methods

Seventy-four arterial blood samples were analysed with three POC devices (Cobas, Roche (CO); ABL800 Flex, Radiometer (ABL); Gem Premiere, Instrumentation Laboratory (IL)). For selected parameters, samples were also analysed in the central laboratory. pCO_2_, pO_2_, SO_2_, bicarbonate and standard bicarbonate (HCO_3 _and HCO_3_std), sodium, potassium, calcium, pH, lactate, base excess (BE(B) and BEecf), glucose, hemoglobin and hematocrit were compared.

## Results

For most parameters only minor, although statistically significant, changes were observed between the POC devices. For pO_2_, BE(B), hemoglobin and hematocrit, clinically significant differences were found. When for example looking at a pO_2 _of 60 mmHg, in six out of 74 samples, IL and/or CO showed a pO_2 _below 60 mmHg and ABL showed a pO_2 _of above 60 mmHg. For hematocrit and hemoglobin, differences between the devices would result in different decisions regarding the use of packed red cells in 11 to 19% of the samples. For BE(B) in a total of 15% of measurements, the results obtained from the different devices would not agree whether a BE(B) is normal or not.

## Conclusions

Although POC devices are of high standard and overall comparability between devices is high, there might be a clinically relevant bias between devices, as found in our study for pO_2_, BE(B), hemoglobin and hematocrit. This can be of importance when interpreting results of the same patient obtained from different POC devices, as could happen when a patient is transferred within a hospital where different devices are used.

